# Avocado Seed: A Comparative Study of Antioxidant Content and Capacity in Protecting Oil Models from Oxidation

**DOI:** 10.3390/molecules23102421

**Published:** 2018-09-21

**Authors:** Francisco J. Segovia, Gádor Indra Hidalgo, Juliana Villasante, Xavier Ramis, María Pilar Almajano

**Affiliations:** 1 Chemical Engineering Department, Universitat Politècnica de Catalunya, Av. Diagonal 647, 08028 Barcelona, Spain; segoviafj@gmail.com (F.J.S.); chemicontact@gmail.com (G.I.H.); julianavillasante@gmail.com (J.V.); 2 Heat Engines Department, Universitat Politècnica de Catalunya, Av. Diagonal 647, 08028 Barcelona, Spain; ramis@mmt.upc.edu

**Keywords:** avocado seed, EPR, radical scavenging, oxidation induction time, differential scanning calorimetry, emulsion, antioxidant, oil, lipid peroxidation

## Abstract

Increasingly, consumers want products containing little or no synthetic compounds. Avocado seeds, which are a residue of the food industry, could be used to obtain extracts with high antioxidant power. In the present study, the most popular radical scavenging methods are presented, establishing a comparison between them, besides working with two different extractions: pure methanol and ethanol–water (50:50 *v/v*). The radical scavenging assay methods ORAC and ABTS were performed, as well as a novel method: the reaction to methoxy radical, as determined by electron paramagnetic resonance (EPR). Peroxide value and thiobarbituric acid reactive compounds (TBARs) were used to monitor the oxidation of avocado seed oil, as well as the power of the avocado seed extract (ASE) to delay oil oxidation by oxidation induction time (OIT) and measured by differential scanning calorimetry (DSC). Radical scavenging methods have values between 1310–263 µmol TE/g of mass dissolved for ORAC and ABTS, respectively. The individual contribution of each of the compounds present in the extract was analyzed. The sum of all of them contributed up to 84% of the total radical scavenging activity. The concentration of 0.75% ASE causes a delay in the oxidation that is close to 80%, as measured by OIT. This implies that avocado seed residue may have a use as a natural antioxidant source, providing added value to organic waste.

## 1. Introduction

Polyphenols are widely recognized to have antioxidant properties [1]. They are very useful in food preservation to extend the shelf life of products, since they protect against microorganisms and prevent lipid peroxidation due to the attack of free radicals [2,3]. In addition, they protect against direct or indirect oxidation caused by metal cations [4]. These cations stimulate the creation of reactive oxygen species (ROS), which are harmful to human health. Previous to human studies, the determination of the radical scavenging (ORAC, ABTS, and DPPH methods) are commonly applied for the analysis of many matrices as vegetables [5,6,7,8], juice fruits [9,10], and vegetables oils [11,12].

The food industry elaborates many by-products and waste due to its normal fabrication of goods. These types of residues have a significant environmental impact due to the great organic charge they contain, as well as their associated handling, transport, and storage costs, among others [13,14]. Therefore, more alternative uses for these wastes are sought, as, for instance, animal feed and fertilizers. Some examples of these [15,16,17] are found in the fruit juice industry, where a large amount of oil, skin, and seeds from oranges, apples, and peaches are wasted with a high content of polyphenols. Also, the residues from wine production include phenolic compounds [18]. Giuffrè showed that the amounts of phenolic compounds that were contained in grape skin changed throughout the fruit-ripening process [19]. Other studies have focused on the shells of nuts, in which large amounts of tannins are found. There is evidence that the skin may even have a greater amount of polyphenols than the kernel itself [20]. These by-products are used in the chemistry, cosmetic, and pharmaceutical industries as natural additives [13].

The avocado (*Persea americana* Mill.) is native to Central America. Mexico is the largest producer of avocados worldwide [21], and the worldwide production exceeds three million tons. In the industry, the pulp is used, while the skin and the seeds are discarded. These residues are rich in polyphenols with antioxidant and antimicrobial power [22]. Condensed tannins, phenolic acids, and flavonoids were the most representative groups in avocado seed. Among the polyphenols, (+)-catechin, (−)-epicatechin, and 3-leucoanthocyanidins are found [23]. Recent studies demonstrated that the seed of this fruit presents anti-inflammatory and anti-carcinogenic properties [24].

It has also been observed that the ASE presents acetogenin, with antimicrobial effect. These properties are stable in complex food systems [25], being useful in preventing the oxidation of model food systems such as emulsions of sunflower oil in water (10% oil) or meat burgers. They have been shown to be effective in preventing oxidation and microbial growth [26]. In both cases, it has been found that they can slow down oxidation over 60% [27].

The goal of this work is wide: (a) to compare different traditional methods of radical scavenging, including the EPR methodology with the real-methoxy radical, (b) to analyze the contribution of each of the components present in the residue, and (c) to compare different techniques where the oxidation of sunflower oil is forced, including the OIT (oxidation induction time).

Avocado seed extract is effective as a natural antioxidant, showing protection against oxidation when added to sunflower oil as a food model. The main antiradical activity is due to several antioxidant molecules such as polyphenols and flavonoids. 

## 2. Results and Discussion

### 2.1.Total Polyphenol Content (TPC) and Radical Scavenging Activity

There are various authors working with different extracts from avocado seeds. Table 1 shows the conditions of extraction and the results obtained. The conversion to the same units was done to enable easy comparisons. There is a large scatter in the results, which is difficult to justify. On the one hand, the highest value obtained is in the extraction performed with methanol/water (75:25) by Pahua Ramos [28], while the lower value with the same kind of avocado (*Persea americana* Mill. var. Hass) is obtained in the extraction with methanol/water 80:20 at 60 °C, which is 30 times lower than the value obtained by Kosinska [29]. On the other hand, López worked with *Persea schiedeana,* a different species from the same Genus than the avocado, and found lower values [30]. In the present study, the TPC that was found was 30.98 ± 0.68 mg gallic acid equivalents (GAE)/g DW with ethanol/water 50:50 extraction at 4 °C overnight. It is similar to the amount described by Rodríguez-Carpena [31] with methanol/water 70:30. In this research, ethanol:water (50:50) was selected due to its Generally Recognized As Safe (GRAS) nature versus methanol.

In Table 1, four authors reported the results for the ORAC, and the dispersion was similar. Results range from above 600 µmol TE/g DW [27] when extracting with ethanol down to those values close to 100 µmol TE/g DW [35] when using methanol. The value obtained in the present study was the highest, which was more than 1300 µmol TE/g DW. No correlation between the two values (ORAC/TPC) was found for any of the displayed articles.

Using the ABTS as a radical, the dispersion is also remarkable. The highest value [26] is greater by a factor of almost 30 times to the one obtained for the different species, *Persea schiedeana* [30]. The value obtained in this study is in the lower range for *Persea americana* (263 µmol TE/g DW). Apparently, here, the use of ethanol as the extracting solvent yields higher values of antioxidant capacity with ABTS. Regarding DPPH, high variability was also reported amongst studies, concluding that the extraction conditions, the sample origin, and the methodology for radical scavenging determination affect the results obtained to a great extent.

In the present study, the most popular radical scavenging methods are presented, establishing a comparison between them, besides working with two different extractions. On the other hand, a novel method is incorporated, the reaction to methoxy radical, as determined by EPR. Table 2 shows the results with different units. The EPR analyzes a competitive reaction to DMPO [36,37,38], which acts also, scavenging the radicals generated “in situ”.

Table 2 shows the radical scavenging values obtained with pure methanol and ethanol/water (50:50). There is not a value for the EPR with ethanol/water, because there is an interference with the water in the determination. The standard used is ferulic acid for a similar reason, in order to avoid interferences and facilitate the solubility in the adequate concentration.

For the EPR determination, the value for the ASE is lower than the other values obtained previously by Azman et al. in white tea (1.33 ± 0.3 FAE/g white tea extract) [38].

In plant extracts, products are many individual compounds contributing to the overall antioxidant activity. While we would highlight the interactions between them and the synergistic effect, it is also important to consider the individual contribution to the antioxidant activity exerted by the compounds separately. To do this, after the separation by HPLC with a gradient polarity (as it is contained in materials and methods), the injection of the ABTS radical generated “in situ” was performed. Therefore, the negative peak corresponds to the radical scavenging activity, and the higher negative area corresponds to the higher antiradical activity.

**Figure 1:**
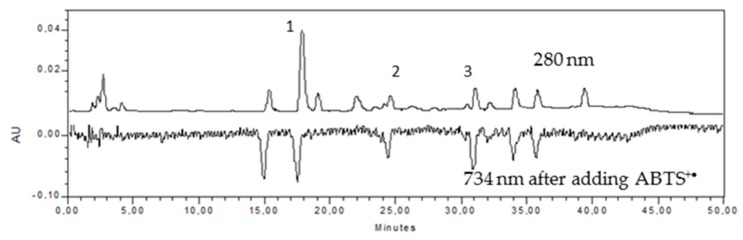
HPLC chromatogram of the extract of ASE. Chlorogenic acid (1), (+)-Catechin (2), (−)-Epicatechin (3).

In direct chromatogram, prior to the injection of ABTS, (+)-catechin, (−)-epicatechin, and an isomer of chlorogenic acid have been identified at concentrations of 20.10 mg/L extract, 27.89 mg/L extract, and 51.59 mg/L extract, respectively. In addition, there are three peaks belonging to the family of flavonoids. Rodríguez-Carpena [26] and Kosinska [29] also found these compounds in amounts of 57.5 µg/g DW and 282.7 mg/100 g DW. Figure 1 shows the HPLC performed to ASE. The chlorogenic acid is the polyphenol that is found in highest quantity. This acid is found in many natural plant extracts, and its influence on the antioxidant capacity and captured hydroxyl radicals has been amply demonstrated [39], because it contains a catechol group that makes it especially effective for capturing free radicals [40,41].

Figure 1 also includes the “negative” peaks, which are in the chromatogram having antiradical activity. This method has already been described in earlier publications, and is an effective, fast, and sensitive analysis for individual components [42,43,44]. Table 3 lists the values quantified with gallic acid.

The first flavonoid (RT 15.36 min) with the chlorogenic acid and the (−)-epicatechin are those that provide the largest percentage of radical scavenging activity. Below are (+)-catechin and other flavonoids. The sum of the percentages of the individual peaks provides over 84% of the radical scavenging activity with the ABTS radical. The difference up to 100% can be due to the synergistic effect between the different compounds or also due to other compounds that are not detectable with the separation by this HPLC method. In any case, the percentage of individual peaks is very high, and justifies the major part of the antiradical activity. All of them are well-known antioxidants that are present in coffee, tea, and other plant extracts [45,46,47,48], which have been successfully used in preventing lipid oxidation in foods [38,49,50].

### 2.2. Protective Effect of ASE in Sunflower Oil Fatty Acid Mixture

To evaluate the antioxidant activity in a model system (sunflower oil stripped from its natural antioxidants), different percentages of ASE were added. The control sample was prepared without antioxidant, and the positive controls were prepared with synthetic antioxidant (BHT). These samples were subjected to two types of analysis. The evolution of primary oxidation was monitored by peroxide value (PV) and secondary oxidation by thiobarbituric acid reactive compounds (TBARs). The first forced oxidation was carried out at moderate temperature (23 days at 35 °C). The second method is a much forced oxidation, which determines the OIT (oxidation induction time) by DSC (differential scanning calorimetry) at 100 °C and 10 L/min of air.

#### 2.2.1. Primary Oxidation of Sunflower Oil by Peroxide Value

**Figure 2:**
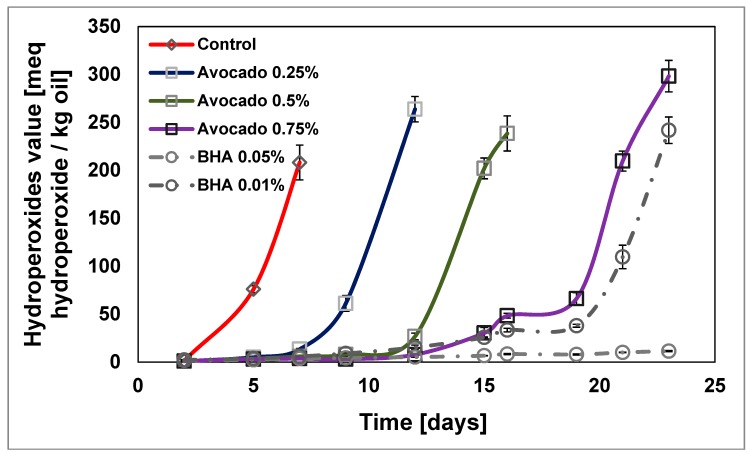
Changes in the peroxide value (PV) of a sunflower oil fatty acid mixture at 35 °C.

The evolution of PV results over time in moderate oxidation (35 °C) is set out in Figure 2. In addition, Table 4 lists the induction time of each and the slope or rate of oxidation at the starting time. Vaidya showed similar results working with walnut oil and grape seed oil [51]. Three different concentrations (0.25%, 0.50% and 0.75%) of ASE have been studied. The effect on the antioxidant activity is proportional to the concentration. Furthermore, in this range of concentrations, it has not reached a concentration that can have pro-oxidant effect.

For the concentration of 0.75% of ASE, the maximum PV is reached at t = 500 h, which compared to the same value for the control (t = 100 h), implies a reduction of oxidation, with a more than five times increased shelf life. The results are similar to those obtained with 0.01% BHA. The lower concentration of ASE values (0.25%) also has a protective effect, although lower (two times delayed with respect to the control sample). Abdelazim [52] worked with sesame extract and found a similar delay of the oxidation.

Other natural sources of antioxidants from food have been previously compared with synthetic preservatives. Sesame cake extract at a concentration of 200 mg/L has stabilization efficiency comparable to commonly-used synthetic antioxidants BHT and BHA at their legal limit, but has lower efficiency than that of the synthetic antioxidant TBHQ [52]. Also, it has been successfully worked with pure compounds as chlorogenic acid and caffeic acid in the presence of mixtures triacylglycerols, and they found a similar delay in the samples oxidation, where at 2.8 × 10^−4^ M, both acids show equal effectiveness and strength. At concentrations above 10 × 10^−4^ M, caffeic acid appears as a much more effective and stronger inhibitor [45].

The control has a negligible induction time (Table 4). All of the other samples have an induction time that allows the calculation of PV_10_. This is a distinguishing feature to previous studies [53], demonstrating the high antioxidant activity of ASE at the concentrations used.

#### 2.2.2. Secondary Oxidation of Sunflower Oil by TBARs

**Figure 3:**
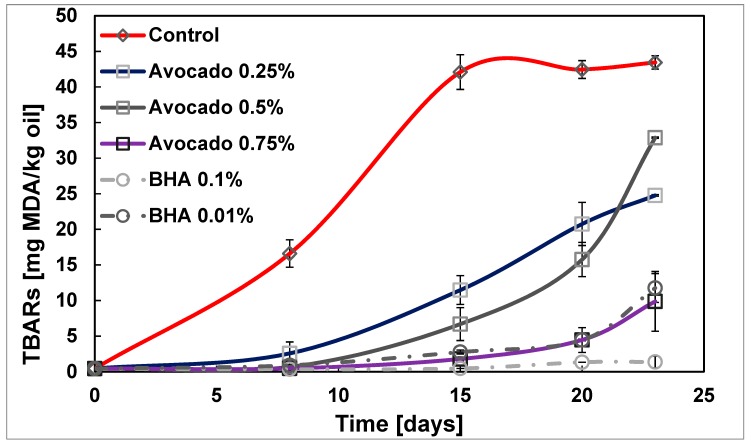
Changes in the TBARs value of sunflower oil fatty acid mixture at 35 °C in the dark.

Figure 3 shows the evolution of TBARs over time. The first values (at the beginning of the oxidation, before the hydroperoxides have been formed) are negligible, but the increase starts at the eighth day which coincides with a significant increase in the compounds obtained by the primary oxidation. The sample with 0.25% of ASE has a delay of 43% over the control one, while in the sample with 0.75%, the percentage of delay is 77%.

**Figure 4:**
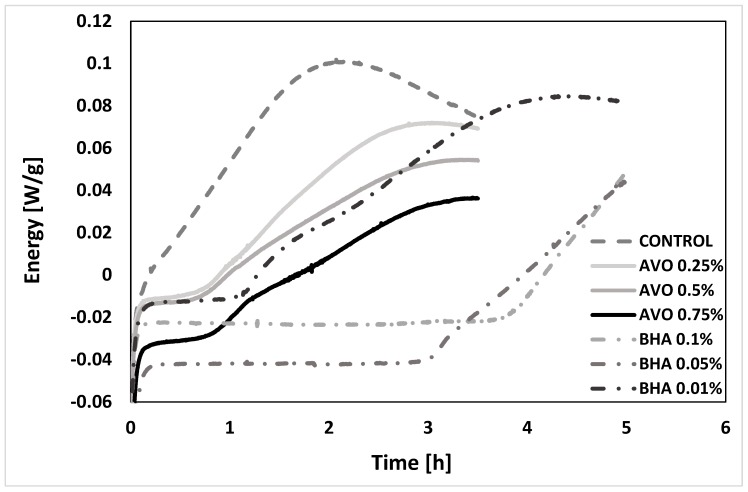
Isothermal analysis to determinate OIT value for sunflower oil fatty acid mixture.

The OIT method allows the obtainment of comparable results in hours versus days of the PV and TBARs. It is to perform an accelerated oxidation with oxygen in the conditions studied and measured by DSC. They are shown in Figure 4 and Table 4. It is not the first time that this method has been used to calculate the oxidation. For example, it was used in a study with cocoa butter [54] with added extracts of pulp obtained from Barbados cherry, mango, and guava, which increased the oxidative stability of soybean oil at certain concentrations [55]. It was also used to determine the time that it takes to oxidize a fat, and consequently the protective effect that the extract or antioxidant that was added had in other oils such as cottonseed, canola, and sunflower [56], with good results. The considerable reduction of time (less than 6 h per sample) allows it to be a quick and reliable method for the food industry, to assess the protective effect of antioxidants or, where applicable, to find potential synergies that may decrease the final amount of a particular synthetic antioxidant.

In Figure 4, the control displays an immediate oxidation in the conditions applied, where the OIT is very difficult to appreciate at this scale. No significant differences were found between the two lower concentrations of ASE used. Both have an OIT somewhat higher 41–43 min, representing more than 45% of the protection against the forced oxidation compared to the control. Nevertheless, in the higher concentration (0.75%), the OIT has a value close to 53 min, which is above the value achieved with the lower concentration of BHA (0.01%), and an increase of 85% in the stability of the fatty acid mixture analyzed (Table 4). As in other studies where they have applied natural extracts to prevent the oxidation of fish oils [57], sunflower oil that is high in oleic acid and castor oil natural extracts prevents oxidation; [58] the ASE possesses antioxidant activity due to the influence of the components found in the extract.

Future research could be focused on the application of ASE into foods such as meat, fish, margarine, etc., and quantify the increase in the shelf life of those foods, as well as asses the acceptability of the products in the market by sensory analysis.

## 3. Materials and Methods

### 3.1. Sample and Extracts Preparation

Refined sunflower oil was purchased from a local retail outlet. Sunflower oil was passed through alumina as described by Skowyra [59] in order to remove naturally present tocopherols. The avocado (*Persea americana* Mill. var. Hass) was obtained from a local market in Barcelona (Spain), with the adequate ripeness for consumption; the seeds were separated from other edible parts. First, 25 seeds were ground into a powder by using a Moulinex mill (A5052HF, Moulinex, Lyon, France). The particle size was standardized with a number 40 mesh sieve. It was homogenized and frozen at −80 °C for lyophilization. Finally, the powder was stored in an amber bottle in a desiccator until use.

Extraction was carried out in amber bottles. Lyophilized powder (0.25 g) was blended with 25 mL of solvent (methanol/water 50:50). This mixture was placed under stirring in a refrigerator at 4 °C overnight, centrifuged (Orto Alresa, Madrid, Spain) at 2500 rpm for 10 min, and the supernatant was separated as extract. Ethanol was eliminated by rotoevaporation, and the extract was freeze-dried and stored until used for analysis. To obtain the sample for EPR, pure methanol was used.

### 3.2. Material and Reagents

Trolox (6-hydroxy-2,5,8-tetramethylchroman-2-carboxylic acid), ethanol, fluorescein, AAPH, BHA, and 2-thiobarbituric acid, Chlorogenic acid, (+)-Catechin, and (-)-Epicatechin were purchased from Sigma-Aldrich Company Ltd. (Gillingham, UK). Folin-Ciocalteu reagent, sodium carbonate and 1,6-diaminohexane were supplied by Merck (Darmstadt, Germany). Iron(II) sulfate (FeSO_4_), DMPO, H_2_O_2_, MeOH, trichloroacetic acid, and hydrochloric acid were acquired from Panreac Química S.L.U. (Barcelona, Spain). All of the compounds were of reagent grade.

### 3.3. Chemical Analysis

#### 3.3.1. Total Polyphenol Content (TPC)

TPC was determined spectrophotometrically following the Folin–Ciocalteu colorimetric method [60,61]. A sample diluted 1:4 with milli-Q water was stirred in triplicate. The final concentration in each one of the 96-well plates that were used was: 7.7% *v/v* sample, 4% *v/v* Folin–Ciocalteu’s reagent, 4% saturated sodium carbonate solution, and 84.3% of milli-Q water, all mixed. The solution was allowed to react for 1 h in the dark, and the absorbance was measured at 765 nm using a Fluostar Omega (BMG Labtech, Ortenberg, Germany). The total phenolic content was expressed as mg gallic acid equivalents (GAE)/g dry weight.

#### 3.3.2. Radical Scavenging Activity

ORAC [27], FRAP [62], and TEAC [63] methods were used. The results are expressed as µmol Trolox equivalents (TE)/g of dry weight or µmol ferulic acid equivalents/g of dry weight.

#### 3.3.3. Determination of Methoxy Radical Scavenging Activity by EPR

The method was reported by Azman [38]. The extract was prepared in deoxygenated MeOH. A spin-trapping reaction mixture that consisted of 100 μL of DMPO (35 mM), 50 μL of H_2_O_2_ (10 mM), 50 μL of avocado methanol extract (0−8.13 g/L), or 50 μL of ferulic acid used as reference (0−20 g/L) or 50 μL of pure MeOH used as a control; and, finally, 50 μL of FeSO_4_ (2 mM). The final solutions (250 μL) were passed through a narrow quartz tube (inside diameter = 2 mm) and introduced into the cavity of the EPR spectrometer. The spectrum was recorded 12 min after the addition of the FeSO_4_ solution, when the radical adduct signal was the greatest.

X-band EPR spectra were recorded with a Bruker EMX-Plus 10/12 spectrometer (Bruker, Billerica, MA, USA) under the following conditions: microwave frequency, 9.876 GHz; microwave power, 30.27 mW; center field, 3522.7 G; sweep width, 100 G; receiver gain, 5.02 × 104; modulation frequency, 100 kHz; modulation amplitude, 1.86 G; time constant, 40.96 ms; and conversion time, 203.0 ms.

Each measurement was carried twice. The first derivative of the absorption signal was integrated in duplicate, resulting in it being directly proportional to the concentration of the remaining radical adducts values, when the competitive reactions of the methoxy radical with DMPO and the antioxidant were completed. Then, these values were compared with those obtained with ferulic acid, which was used as a standard.

#### 3.3.4. High Performance Liquid Chromatography (HPLC)

Identification and quantification were performed using a Waters 2695 Separations Module (Meadows Instrumentation, Inc., Bristol, WI, USA) system with a photodiode array detector Waters 996 (Meadows Instrumentation, Inc.). A Kinetex 2.6-u C18 100A, (100 × 4.6 mm) column was used. Mobile phase was 0.1% acetic acid in water (*v*/*v*) (eluent A) and 0.1% acetic acid in acetonitrile (eluent B). The gradient used was: 0–2 min, isocratic gradient from 0% B; 2–40 min, linear gradient from 0–15% B; and a 40–50 min linear gradient from 0–15%. B. The flow rate was 0.8 mL/min. Detection wavelengths were 280 nm and 330 nm. The sample injection volume was 10 µL. The chromatographic peaks were confirmed by comparing their retention times and diode array spectra against those of their reference standards, and the chlorogenic acid was confirmed by MS HPLC-MS. Working standard solutions between 100–500 mg/L were injected into the HPLC to obtain the calibration curve plotting concentration (mg/L) versus area. Quantification was carried out from integrated peak areas of the samples using the corresponding standard graph.

For the analysis of the radical scavenging activity of each of the compounds by ABTS radical, a pump Merk-Hitachi HPLC gradient pump (Model L-6200) (Hitachi High Technologies America Inc., Schaumburg, IL, USA) was coupled with a 0.2 mL/min flow with an ABTS concentration of 0.03% (*w*/*v*). This allowed a perfect mixture thanks to the 3 m of tube where the flow could homogenize before reaching the detector.

The reading wavelength was 734 nm. The calibration curve to quantify the results was made with Gallic acid.

#### 3.3.5. Determination of Primary Oxidation with Peroxide Value (PV) and Secondary Oxidation with TBARs Method

PV was determined by the ferric thiocyanate method [64] (after calibrating the procedure with a series of oxidized oil samples analyzed using the AOCS Official Method Cd 8-53 [65]). Data from the PV measurements were plotted against time.

The secondary oxidation of oil was determined by the concentration of thiobarbituric acid reactive substances (TBARs) using the method described by Gallego [66] with slight modifications. An amount of each sample was taken and the TBARs reagent (15% trichloroacetic acid, 0.375% thiobarbituric acid and hydrochloric acid 2.1%) was added in a ratio 1:5. Immediately, the samples were introduced in an ultrasonic bath (Prolabo brand equipment, Rue des Casernes, Sion) and immersed in a water bath pre-heated to 95 °C. Samples were centrifuged, and the absorbance of the supernatant was measured at λ = 531 nm. The results are expressed as mg MDA/kg of oil.

#### 3.3.6. Oxidation Induction Time analysis (OIT-DSC)

Differential scanning calorimetry (DSC) experiments were performed with a DSC 820 from Mettler Toledo (Schwerzenbach, Switzerland) under isothermal conditions (100 °C) and with an airflow of 10 mL/min. The samples (5.00 ± 0.25 mg) were weighed into 40 µL aluminum DSC open crucible in order to allow the oil to be in contact with the oxygen stream. An empty crucible was used as reference.

## 4. Conclusions

The ASE is effective as a natural antioxidant. The main antiradical activity is due to polyphenols (+)-catechin, (−)-epicatechin, 3-*O*-caffeoylquinic acid (chlorogenic acid isomer), and three compounds of the flavonoid family. Its individual activity has been demonstrated by the HPLC post-column injection of ABTS, by different radical scavenging methods, including EPR, and also in the protection of sunflower oil oxidation devoid of its natural antioxidants. The degree of oxidation was followed by traditional methods (PV, TBARs), and also was determined by the OIT. Both are correlated, which states that it could be determined only by the OIT, saving both materials and time.

## Figures and Tables

**Table 1:** Different radical scavenging values found in the bibliography.

Seed Sample	Extraction Conditions	TPC [mg GAE/g DW]	ORAC [µmol TE/g DW]	ABTS [µmol TE/g DW]	DPPH [µmol TE/g DW]	FRAP [µmol TE/g DW]	Ref.
*Persea schiedeana*	Acetone/water/acetic acid (70:29.7:0.3, *v/v/v*), r.t.	6.15 ± 0.08	-	58.45 ± 1.39	37.64 ± 4.81	42.81 ± 0.80	[30]
*Persea americana* var. Hass	Acetone/water/acetic acid (70:29.7:0.3, *v/v/v*), r.t.	16.5	137.2	-	52.7	-	[32]
*Persea americana* var. Hass	Ethanol/water (1:1, *v/v*), 200 °C, 11 MPa	-	310 ± 30	300 ± 20	15 ± 2 ^γ^	-	[23]
*Persea americana*	Ethanol/Water (1:1, *v/v*), 70 °C	88.2 ± 2.2	-	725 ± 39.4	-	1484 ± 15.7	[33]
*Persea americana*	Ethanol/water (56:44, *v/v*), 63 °C	45.01	616.48	-	-	-	[27]
*Persea americana* var. Hass ^1^ and Fuerte ^2^	Ethanol/Water (80:20, *v/v*), r.t.	57.3 ± 2.7 ^1^, 59.2 ± 6.9 ^2^	-	645.8 ± 17.9 ^1^, 580.8 ± 31.0 ^2^	410.7 ± 35.8 ^1^, 464.9 ± 32.7 ^2^	656.9 ± 26.0 ^1,^^δ^, 931.7 ± 65.6 ^2,^^δ^	[34]
*Persea americana* var. Hass	Methanol/Water (80:20, *v/v*), 60 °C	9.51 ± 0.161	210	94	-	-	[29]
*Persea americana*	Soxhlet Methanol/water (75:25, *v/v*)	292.00 ± 9.81	-	173.3	-	-	[28]
*Persea americana* var. Hass ^1^ and Fuerte ^2^	Methanol/water (70:30, *v/v*), r.t.	35.11 ^1^, 41.64 ^2^	-	78.93 ± 26.73 ^1,^^β^, 121.61 ± 31.87 ^2,^^β^	66.24 ± 24.84 ^1,^^β^, 94.27 ± 30.47 ^2,^^β^	CUPRAC: 141.67 ± 41.24 ^1,^^β^, 184.42 ± 66.05 ^2,^^β^	[31]
*Persea americana* var. Hass ^1^ and Fuerte ^2^	Acetone/water (70:30, *v/v*), r.t.	60.82 ^1^, 69.12 ^2^	-	158.29 ± 26.27 ^1,^^β^, 194.80 ± 44.69 ^2,^^β^	130.26 ± 36.80 ^1,^^β^, 167.50 ± 42.08 ^2,^^β^	CUPRAC: 275.36 ± 59.09 ^1,^^β^, 353.43 ± 75.83 ^2,^^β^	[31]

^α^ [µmol TE/g DW]; ^β^ [mmol TE/g FW]; ^γ^ IC_50_[µg/mL]; ^δ^ [µmol Fe^2+^/g DW]; ^1^ var. Hass; ^2^ var. Fuerte.

**Table 2:** Radical scavenging values of avocado seed extract (ASE) obtained with pure methanol and ethanol/water.

Method	Methanol, 4 °C, 24 h	Ethanol/water (50:50, *v/v*) 4 °C, 24 h
TPC [mg GAE/g DW]	25.35 ± 0.77	30.98 ± 0.68
ORAC [µmol TE/g DW]	1240 ± 70 (0.59 ± 0.03 ^1^)	1310 ± 40
ABTS [µmol TE/g DW]	123.74 ± 2.46 (0.15 ± 0.00 ^1^)	263.58 ± 17.85
FRAP [µmol TE/g DW]	316.60 ± 6.87 (0.19 ± 0.00 ^1^)	438.89 ± 7.32
EPR [µmol FAE/g DW]	0.53 ± 0.07	-

^1^ g ferulic acid equivalents (FAE)/g DW.

**Table 3:** ASE composition and antioxidant capacity of their compounds.

Name	Retention Time (RT)	HPLC Peak Area	Concentration mg/L	HPLC-ABTS Peak Area	%	Antioxidant Capacity [mg GAE/L]	% Antioxidant Activity in the Total Extract
Procyanidin 1 *	15.36	199,682	-	1,956,638	21.76	53.12	16.3
Chlorogenic acid	17.88	1,011,205	51.59	1,901,135	21.14	51.86	16.0
(+)-Catechin	24.59	157,538	20.10	977,869	10.88	30.84	9.5
(−)-Epicatechin	31.07	233,557	27.89	1,574,801	17.51	44.43	13.7
Procyanidin 2 *	32.21	80,373	-	265,837	2.96	14.63	4.5
Procyanidin 3 *	34.12	216,547	-	1,169,588	13.01	35.21	10.8
Procyanidin 4 *	35.82	192,750	-	960,683	10.68	30.45	9.4
Catechin 1 **	39.40	205,953	-	184,943	2.06	12.79	3.9

* belongs to the family of procyanidin; ** belongs to the family of catechins.

**Table 4:** Parameters of the different methods to calculate the antioxidant activity.

Sample	IT [days] ^1^	OIT [min] ^2^	PV_10_ [meq hydroperoxide/kg oil] ^3^	PV Slope [meq Hydroperoxide/kg oil·days]	TBARs_15_ [mg MDA/kg oil] ^4^	TBARs Slope [mg MDA/kg oil·days]
Control	5.00	28.51	-	66.08	42.09 ± 1.92 ^a^	3.64
Avocado 0.25%	8.31	41.55	110.03 ± 13.28 ^a^	67.52	11.47 ± 1.60 ^b^	1.27
Avocado 0.5%	11.65	43.22	26.74 ± 3.50 ^b^	54.21	6.69 ± 1.04 ^b^	0.86
Avocado 0.75%	18.85	52.54	7.73 ± 0.98 ^c^	58.02	1.79 ± 0.38 ^c^	0.19
BHA 0.01%	19.17	4.54	15.37 ± 2.28 ^d^	51.00	2.76 ± 0.08 ^d^	0.26
BHA 0.05%	-	127.79	5.23 ± 0.19 ^e^	-	0.99 ± 0.00 ^e^	0.06

^1^ Data from PV graphics; ^2^ Data from differential scanning calorimetry (DSC) graphics; ^3^ Hydroperoxide value at 10 days of experiment; ^4^ Thiobarbituric acid reactive compounds (TBARs) value at 15 days of experiment. ^a,b,c,d,e^ Means within each column with different superscripts are significantly different (*p* < 0.05). IT: induction time; OIT: oxidation induction time.

## Abbreviations

TPC, Total Polyphenol Content; EPR, Electron Paramagnetic Resonance; ABTS, 2,2′azino-bis(3-ethyl-benzothiazoline-6-sulfonicacid); Trolox, (±)-6-hydroxy-2,5,7,8–tetramethylchromane-2-carboxylic acid; GAE, gallic acid equivalents; DW, dry weight; HPLC, High Performance Liquid Chromatography; TEAC, Trolox Equivalent Antioxidant Capacity; TE, Trolox Equivalent; ORAC, Oxygen Radical Absorbance Capacity; FRAP, Ferric Reducing Ability of Plasma; VP, Peroxide Value; TBARs, Thiobarbituric Acid Reactive substances; OIT, Oxidation Induction Time; DMPO, 5,5-dimethyl-pyrroline N-oxide; BHA, Butylated hydroxyanisole; BHT, Butylated hydroxytoluene; r.t., room temperature.

## References

[1] King A.J., Griffin J.K., Roslan F. (2014). In vivo and in vitro addition of dried olive extract in poultry. J. Agric. Food Chem..

[2] Perumalla A.V.S., Hettiarachchy N.S. (2011). Green tea and grape seed extracts—Potential applications in food safety and quality. Food Res. Int..

[3] Jordán M.J., Lax V., Rota M.C., Lorán S., Sotomayor J.A. (2012). Relevance of carnosic acid, carnosol, and rosmarinic acid concentrations in the in vitro antioxidant and antimicrobial activities of *Rosmarinus officinalis* (L.) methanolic extracts. J. Agric. Food Chem..

[4] Wettasinghe M., Shahidi F., Amarowicz R., Abou-Zaid M.M. (2001). Phenolic acids in defatted seeds of borage (*Borago officinalis* L.). Food Chem..

[5] Thaipong K., Boonprakob U., Crosby K., Cisneros-Zevallos L., Hawkins Byrne D. (2006). Comparison of ABTS, DPPH, FRAP, and ORAC assays for estimating antioxidant activity from guava fruit extracts. J. Food Compos. Anal..

[6] Apak R., Güçlü K., Demirata B., Özyürek M., Çelik S.E., Bektaşoǧlu B., Berker K.I., Özyurt D. (2007). Comparative evaluation of various total antioxidant capacity assays applied to phenolic compounds with the CUPRAC assay. Molecules.

[7] Yashin A., Yashin Y., Wang J.Y., Nemzer B. (2013). Antioxidant and antiradical activity of Coffee. Antioxidants.

[8] Shin D., Chae K.S., Choi H.R., Lee S.J., Gim S.W., Kwon G.T., Lee H.T., Song Y.C., Kim K.J., Kong H.S. (2018). Bioactive and pharmacokinetic characteristics of pre-matured black raspberry, *Rubus occidentalis*. Ital. J. Food Sci..

[9] Cano-Lamadrid M., Hernández F., Nowicka P., Carbonell-Barrachina A.A., Wojdyło A. (2018). Formulation and storage effects on pomegranate smoothie phenolic composition, antioxidant capacity and color. LWT.

[10] Lubinska-Szczygieł M., Różańska A., Namieśnik J., Dymerski T., Shafreen R.B., Weisz M., Ezra A., Gorinstein S. (2018). Quality of limes juices based on the aroma and antioxidant properties. Food Control.

[11] Giuffrè A.M., Zappia C., Capocasale M. (2017). Effects of high temperatures and duration of heating on olive oil properties for food use and biodiesel production. J. Am. Oil Chem. Soc..

[12] Giuffrè A.M., Capocasale M., Zappia C., Poiana M. (2017). Influence of high temperature and duration of heating on the sunflower seed oil properties for food use and bio-diesel production. J. Oleo Sci..

[13] Ayala-zavala J.F., Vega-vega V., Rosas-domínguez C., Palafox-carlos H., Villa-rodriguez J.A., Siddiqui M.W., Dávila-Aviña J.E., González-Aguilar G.A. (2011). Agro-industrial potential of exotic fruit byproducts as a source of food additives. Food Res. Int..

[14] Wijngaard H., Hossain M.B., Rai D.K., Brunton N. (2012). Techniques to extract bioactive compounds from food by-products of plant origin. Food Res. Int..

[15] Lagha-Benamrouche S., Madani K. (2013). Phenolic contents and antioxidant activity of orange varieties (*Citrus sinensis* L. and *Citrus aurantium* L.) cultivated in Algeria: Peels and leaves. Ind. Crops Prod..

[16] Aguedo M., Kohnen S., Rabetafika N., Vanden Bossche S., Sterckx J., Blecker C., Beauve C., Paquot M. (2012). Composition of by-products from cooked fruit processing and potential use in food products. J. Food Compos. Anal..

[17] Wijngaard H.H., Brunton N. (2010). The optimisation of solid-liquid extraction of antioxidants from apple pomace by response surface methodology. J. Food Eng..

[18] Guerrero M.S., Torres J.S., Nuñez M.J. (2008). Extraction of polyphenols from white distilled grape pomace: Optimization and modelling. Bioresour. Technol..

[19] Giuffrè A.M. (2013). HPLC-DAD detection of changes in phenol content of red berry skins during grape ripening. Eur. Food Res. Technol..

[20] Wang X., Zhao M., Su G., Cai M., Zhou C., Huang J., Lin L. (2015). The antioxidant activities and the xanthine oxidase inhibition effects of walnut (*Juglans regia* L.) fruit, stem and leaf. Int. J. Food Sci. Technol..

[21] Villamil L., Astier M., Merlín Y., Ayala-Barajas R., Ramírez-García E., Martínez-Cruz J., Devoto M., Gavito M.E. (2018). Management practices and diversity of flower visitors and herbaceous plants in conventional and organic avocado orchards in Michoacán, Mexico. Agroecol. Sustain. Food Syst..

[22] Rodríguez-Carpena J.G., Morcuende D., Estévez M. (2011). Avocado by-products as inhibitors of color deterioration and lipid and protein oxidation in raw porcine patties subjected to chilled storage. Meat Sci..

[23] Figueroa J.G., Borrás-Linares I., Lozano-Sánchez J., Segura-Carretero A. (2018). Comprehensive characterization of phenolic and other polar compounds in the seed and seed coat of avocado by HPLC-DAD-ESI-QTOF-MS. Food Res. Int..

[24] Ahmed N., Smith R.W., Henao J.J.A., Stark K.D., Spagnuolo P.A. (2018). Analytical method to detect and quantify Avocatin B in Hass Avocado Seed and pulp matter. J. Nat. Prod..

[25] Pacheco A., Rodríguez-Sánchez D.G., Villarreal-Lara R., Navarro-Silva J.M., Senés-Guerrero C., Hernández-Brenes C. (2017). Stability of the antimicrobial activity of acetogenins from avocado seed, under common food processing conditions, against *Clostridium sporogenes* vegetative cell growth and endospore germination. Int. J. Food Sci. Technol..

[26] Rodríguez-Carpena J.G., Morcuende D., Estévez M. (2012). Avocado, sunflower and olive oils as replacers of pork back-fat in burger patties: Effect on lipid composition, oxidative stability and quality traits. Meat Sci..

[27] Gómez F.S., Sánchez S.P., Iradi M.G.G., Azman N.A.M., Almajano M.P. (2014). Avocado seeds: Extraction optimization and possible use as antioxidant in food. Antioxidants.

[28] Pahua-Ramos M.E., Ortiz-Moreno A., Chamorro-Cevallos G., Hernández-Navarro M.D., Garduño-Siciliano L., Necoechea-Mondragón H., Hernández-Ortega M. (2012). Hypolipidemic effect of Avocado (*Persea americana* Mill) seed in a hypercholesterolemic mouse model. Plant Foods Hum. Nutr..

[29] Kosińska A., Karamać M., Estrella I., Hernández T., Bartolomé B., Dykes G.A. (2012). Phenolic compound profiles and antioxidant capacity of *Persea americana* Mill. peels and seeds of two varieties. J. Agric. Food Chem..

[30] López-Yerena A., Guerra-Ramírez D., Jácome-Rincón J., Espinosa-Solares T., Reyes-Trejo B., Famiani F., Cruz-Castillo J.G. (2018). Initial evaluation of fruit of accessions of *Persea schiedeana* Nees for nutritional value, quality and oil extraction. Food Chem..

[31] Rodríguez-Carpena J.G., Morcuende D., Andrade M.J., Kylli P., Estévez M. (2011). Avocado (*Persea americana* Mill.) phenolics, in vitro antioxidant and antimicrobial activities, and inhibition of lipid and protein oxidation in porcine patties. J. Agric. Food Chem.

[32] Wang W., Bostic T.R., Gu L. (2010). Antioxidant capacities, procyanidins and pigments in avocados of different strains and cultivars. Food Chem..

[33] Soong Y.Y., Barlow P.J. (2004). Antioxidant activity and phenolic content of selected fruit seeds. Food Chem..

[34] Tremocoldi M.A., Rosalen P.L., Franchin M., Massarioli A.P., Denny C., Daiuto É.R., Paschoal J.A.R., Melo P.S., de Alencar S.M. (2018). Exploration of avocado by-products as natural sources of bioactive compounds. PLoS ONE.

[35] Wang T., Jónsdóttir R., Ólafsdóttir G. (2009). Total phenolic compounds, radical scavenging and metal chelation of extracts from Icelandic seaweeds. Food Chem..

[36] Tobolková B., Polovka M., Belajová E., Koreňovská M., Suhaj M. (2014). Possibilities of organic and conventional wines differentiation on the basis of multivariate analysis of their characteristics (EPR, UV-Vis, HPLC and AAS study). Eur. Food Res. Technol..

[37] Mocan A., Crișan G., Vlase L., Crișan O., Vodnar D.C., Raita O., Gheldiu A.M., Toiu A., Oprean R., Tilea I. (2014). Comparative studies on polyphenolic composition, antioxidant and antimicrobial activities of *Schisandra chinensis* leaves and fruits. Molecules.

[38] Azman N.A.M., Peiró S., Fajarí L., Julià L., Almajano M.P. (2014). Radical scavenging of white tea and its flavonoid constituents by electron paramagnetic resonance (EPR) spectroscopy. J. Agric. Food Chem..

[39] Shi J., Gong J., Liu J., Wu X., Zhang Y. (2009). Antioxidant capacity of extract from edible flowers of *Prunus mume* in China and its active components. LWT-Food Sci. Technol..

[40] Silva B.M., Andrade P.B., Valentão P., Ferreres F., Seabra R.M., Ferreira M.A. (2004). Quince (*Cydonia oblonga* Miller) fruit (pulp, peel, and seed) and jam: Antioxidant activity. J. Agric. Food Chem..

[41] Sendra J.M., Sentandreu E., Navarro J.L. (2007). Kinetic model for the antiradical activity of the isolated *p*-catechol group in flavanone type structures using the free stable radical 2,2-diphenyl-1-picrylhydrazyl as the antiradical probe. J. Agric. Food Chem..

[42] Kosar M., Dorman D., Baser K., Hiltunen R. (2004). An Improved HPLC post-column methodology for the identification of free radical scavenging phytochemicals in complex mixtures. Chromatographia.

[43] Shi S., Zhou H., Zhang Y., Jiang X., Chen X. (2009). Coupling HPLC to on-line, post-column (bio) chemical assays for high-resolution screening of bioactive compounds from complex mixtures. TrAC Trends Anal. Chem..

[44] Koleva I.I., Niederla H.A.G., van Beek T.A. (2001). Application of ABTS radical cation for selective on-line detection of radical scavengers in HPLC eluates. Anal. Chem..

[45] Marinova E.M., Toneva A., Yanishlieva N. (2009). Comparison of the antioxidative properties of caffeic and chlorogenic acids. Food Chem..

[46] Dibert K., Cros E., Andrieu J. (1989). Solvent extraction of oil and chlorogenic acid from green coffee part I: Equilibrium data. J. Food Eng..

[47] Bertrand C., Noirot M., Doulbeau S., de Kochko A., Hamon S., Campa C. (2003). Chlorogenic acid content swap during fruit maturation in *Coffea pseudozanguebariae*: Qualitative comparison with leaves. Plant Sci..

[48] Fujioka K., Shibamoto T. (2008). Chlorogenic acid and caffeine contents in various commercial brewed coffees. Food Chem..

[49] Alarcon E., Campos A.M., Edwards A.M., Lissi E., Lopez-alarcon C. (2008). Antioxidant capacity of herbal infusions and tea extracts: A comparison of ORAC-fluorescein and ORAC-pyrogallol red methodologies. Food Chem..

[50] Pilar Almajano M., Carbó R., LóPez Jiménez J.A., Gordon M.H. (2008). Antioxidant and antimicrobial activities of tea infusions. Food Chem..

[51] Vaidya B., Eun J. (2013). Effect of Temperature on Oxidation Kinetics of Walnut and Grape Seed Oil. Food Sci. Biotechnol..

[52] Abdelazim A.A., Mahmoud A., Ramadan-Hassanien M.F. (2011). Oxidative stability of vegetable oils as affected by sesame extracts during accelerated oxidative storage. J. Food Sci. Technol..

[53] Wardhani D.H., Fuciños P., Vázquez J.A., Pandiella S.S. (2013). Inhibition kinetics of lipid oxidation of model foods by using antioxidant extract of fermented soybeans. Food Chem..

[54] Ciftçi O.N., Kowalski B., Göğüş F., Fadiloğlu S. (2009). Effect of the addition of a cocoa butter-like fat enzymatically produced from olive pomace oil on the oxidative stability of cocoa butter. J. Food Sci..

[55] Araújo K.L.G.V., Magnani M., Nascimento J.A., Souza A.L., Epaminondas P.S., Queiroz N., Queiroz N.S., Aquino J., Souza A.G., Costa M.F.C., Souza A.L. (2017). By-products from fruit processing: One alternative antioxidant for use in soybean oil. J. Therm. Anal. Calorim..

[56] Kodali D.R. (2005). Oxidative stability measurement of high-stability oils by pressure differential scanning calorimeter (PDSC). J. Agric. Food Chem..

[57] Nascimento J.A., Arau K.L.G.V., Epaminondas P.S., Souza A.S., Magnani M., Souza A.L., Soledade L.E.B., Queiroz N., Souza A.G. (2013). Ethanolic extracts of *Moringa oleifera* Lam. Evaluation of its potential as an antioxidant additive for fish oil. J. Therm. Anal. Calorim..

[58] Quinchia L.A., Delgado M.A., Valencia C., Franco J.M., Gallegos C. (2011). Natural and synthetic antioxidant additives for improving the performance of new biolubricant formulations. J. Agric. Food Chem..

[59] Skowyra M., Gallego M.G., Segovia F., Almajano M.P. (2014). Antioxidant Properties of *Artemisia annua* Extracts in Model Food Emulsions. Antioxidants.

[60] Segovia F., Lupo B., Peiró S., Gordon M., Almajano M. (2014). Extraction of Antioxidants from Borage (*Borago officinalis* L.) Leaves—Optimization by response surface method and application in oil-in-water emulsions. Antioxidants.

[61] Prior R.L., Wu X., Schaich K. (2005). Standardized methods for the determination of antioxidant capacity and phenolics in foods and dietary supplements. J. Agric. Food Chem..

[62] Skowyra M., Falguera V., Azman N., Segovia F., Almajano M. (2014). The Effect of *Perilla frutescens* extract on the oxidative stability of model food emulsions. Antioxidants.

[63] Azman N., Segovia F., Martínez-Farré X., Gil E., Almajano M. (2014). Screening of antioxidant activity of *Gentian Lutea* root and its application in oil-in-water emulsions. Antioxidants.

[64] Singh G., Maurya S., DeLampasona M.P., Catalan C.A.N. (2007). A comparison of chemical, antioxidant and antimicrobial studies of cinnamon leaf and bark volatile oils, oleoresins and their constituents. Food Chem. Toxicol..

[65] American Oil Chemists’ Society (AOCS) (1997). Peroxide Value (acetic acid-chloroform method). Official Methods and Recommended Practices of the AOCS.

[66] Gallego M.G., Gordon M.H., Segovia F.J., Skowyra M., Almajano M.P. (2013). Antioxidant properties of three aromatic herbs (Rosemary, Thyme and Lavender) in oil-in-water emulsions. J. Am. Oil Chem. Soc..

